# Cell alignment and accumulation using acoustic nozzle for bioprinting

**DOI:** 10.1038/s41598-019-54330-8

**Published:** 2019-11-28

**Authors:** Yannapol Sriphutkiat, Surasak Kasetsirikul, Dettachai Ketpun, Yufeng Zhou

**Affiliations:** Singapore Centre for 3D Printing (SC3DP), School of Mechanical and Aerospace Engineering, Nanyang Technological University, 50 Nanyang Ave., 639798 Singapore, Singapore

**Keywords:** Biomedical engineering, Tissue engineering

## Abstract

Bioprinting could spatially align various cells in high accuracy to simulate complex and highly organized native tissues. However, the uniform suspension and low concentration of cells in the bioink and subsequently printed construct usually results in weak cell-cell interaction and slow proliferation. Acoustic manipulation of biological cells during the extrusion-based bioprinting by a specific structural vibration mode was proposed and evaluated. Both C2C12 cells and human umbilical vein endothelial cells (HUVECs) could be effectively and quickly accumulated at the center of the cylindrical tube and consequently the middle of the printed construct with acoustic excitation at the driving frequency of 871 kHz. The full width at half maximum (FWHM) of cell distributions fitted with a Gaussian curve showed a significant reduction by about 2.2 fold in the printed construct. The viability, morphology, and differentiation of these cells were monitored and compared. C2C12 cells that were undergone the acoustic excitation had nuclei oriented densely within ±30° and decreased circularity index by 1.91 fold or significant cell elongation in the printing direction. In addition, the formation of the capillary-like structure in the HUVECs construct was found. The number of nodes, junctions, meshes, and branches of HUVECs on day 14 was significantly greater with acoustic excitation for the enhanced neovascularization. Altogether, the proposed acoustic technology can satisfactorily accumulate/pattern biological cells in the printed construct at high biocompatibility. The enhanced cell interaction and differentiation could subsequently improve the performance and functionalities of the engineered tissue samples.

## Introduction

Bioprinting, computer-aided bioadditive manufacturing, provides high accuracy on the spatial placement of cells themselves, recapitulating the cell alignment of complex and highly organized native tissues. This technology is emerging as a promising approach for tissue engineering despite its current infancy. Usually, cells suspended uniformly in the medium (e.g. hydrogel) containing biological factors and biomaterials are used as bioink in the cell, tissue or organ printing. Extrusion-based bioprinting provides relatively better structural integrity because of continuous deposition of cylindrical struts and is the most convenient technique in rapid biofabrication.

The intrinsic limitations for current bioprinting are because of the slow cell proliferation and colonization while cells are immobilized within hydrogels and do not spread, stretch, and migrate rapidly to generate the new and mature tissue^1^. Organized cellular alignment is critical to controlling tissue microarchitecture and biological function. A spatial manipulation (e.g. patterning/alignment) of various cells in bioprinting could improve the performance and functionalities of the printed construct, such as the enhanced cell proliferation and differentiation, enhanced viability owning to the large cell seeding density and less mechanical stress experienced in comparison to direct cell manipulation^1^, better mimicking the native architecture of tissue^2^, high mechanical strength with construct compaction, tissue regeneration without scaffolds (e.g. the use of tissue spheroids)^3^, and significantly reduced amount of biomaterials in the use and time for tissue maturation^4^.

Skeletal muscles (striated muscles) are the major parenchymal tissues of the locomotor system. Although mature skeletal myocytes are capable of regeneration after mild physical injuries and degenerations, severe muscular degenerations caused by aging and nutritional disturbances, putrefactive bacterial myositis, severely acute traumatic myositis, and high-dose irradiation may result in non-regenerative myositis^5^. To solve such a problem, a printed muscle sample which can imitate both the structure and function of native muscle tissue is an alternative strategy for the treatment of various muscular diseases and injuries^6^. The control of cell activities is a key modulator during skeletal muscle adaptation and regeneration^7,8^. The fully-differentiated skeletal muscles are patterned as the multi-nucleated syncytia of striated myocyte fascicles aligned parallel to each other^9^. Therefore, adjacent myocytes must be kept close in order to form, elongate, and orientate the fascicles along their major axis during the histogenesis because of the enhanced cell-cell interaction^10,11^. Patterning of cells during the bioprinting is a promising approach. The highly organized C2C12 myoblasts not only mimic the native architecture of muscle tissue but also contribute to load-bearing muscle^2^.

The most critical challenge in tissue engineering and regenerative medicine is the integration of a vascular network. Without vascularization as natural anatomy of blood vessels, an engineered large tissue is not able to have enough nutrients, gas exchange, and metabolic waste removal for maturation during perfusion, which results in low cell viability and malfunction^12^. Despite the efforts to mimic artery-like structures by bioprinting^13,14^ and templating^15,16^, there are limitations on the fabrication of microvessel^16,17^, such as applicable biomaterials, disaggregation and the large interval between cells, the low density of vessel area, the small size of vascular structure, and long fabrication time^13,14,16^. In addition, the paracrine signaling between the endothelial cell constructs or between the endothelial cell and fibroblast may not be sufficient for angiogenesis and vascular maturation if the cell-cell interaction is weak because of the large distance between the endothelial cells^18^.

There are a few studies attempted to assist the cell manipulation during the bioprinting. For example, the magnetic force could align the microparticles’ orientation^19,20^. But this method requires the use of microparticles with specific electromagnetic properties or labeling the cells/proteins with magnetic nanoparticles, which is time-consuming and may cause some toxicity to organisms (dependent on the composition and physicochemical properties)^21^. Electrical manipulation of the conductive and/or dielectric microparticles also requires certain electrical charge properties^22,23^. The high electric field may induce heating, which affects the viability of the mammalian cells. Acoustic manipulation using either bulk or surface acoustic wave has already been developed for various applications, such as microparticles/cells patterning^24–26^, focusing^27,28^, and sorting^29,30^. This approach relies on the discrepancies between the density and compressibility of microparticles/cells and those of surround fluid. Selective manipulation of cells in a high resolution noninvasively exhibits the great potential to regulate cell-cell distances and engineer cellular aggregates. The advantages of an acoustic approach also include simple device fabrication and experimental setup, low power consumption, and good biocompatibility. However, the patterns of standing acoustic wave in the microchannel are pre-determined by the driving frequency of transducer and geometry of microchannel and cannot be adjusted arbitrarily to mimic the complex pattern^31^.

In our previous study, a structural vibration was successfully generated by attaching a piezoceramic plate to the outside wall of a cylindrical glass tube at the desired resonant frequency^32^. Consequently, the microparticles were accumulated quickly and effectively at the position of pressure nodes, around the center of the cylindrical tube, and subsequently the middle of the printed construct. However, its effects on the growth, morphology, differentiation, and orientation of cells have not been investigated to evaluate its performance in bioprinting. The objective of this study is to utilize the acoustic nozzle to accumulate the cells during bioprinting and then to observe the morphological changes of them in the printed gelatin methacryloyl (GelMA) construct during cell culture. Firstly, the resonant frequency of structural vibration of a cylindrical nozzle filled with biological cells was numerically predicted and validated in the experiment. Subsequently, the distribution of biological cells inside the printed GelMA sample without and with acoustic excitation was quantitatively determined and compared. Lastly, the morphological changes in the cells undergone the acoustic excitation were monitored for up to 14 days. The orientation of the C2C12 cells, a skeletal myoblast cell line of *Mus musculus*^33^, and formation of myotube as well as the generation of a neovascular network of human umbilical vein endothelial cells (HUVECs) were evaluated during the cell culture process.

## Results

### Cell accumulation by acoustic excitation

**Figure 1:**
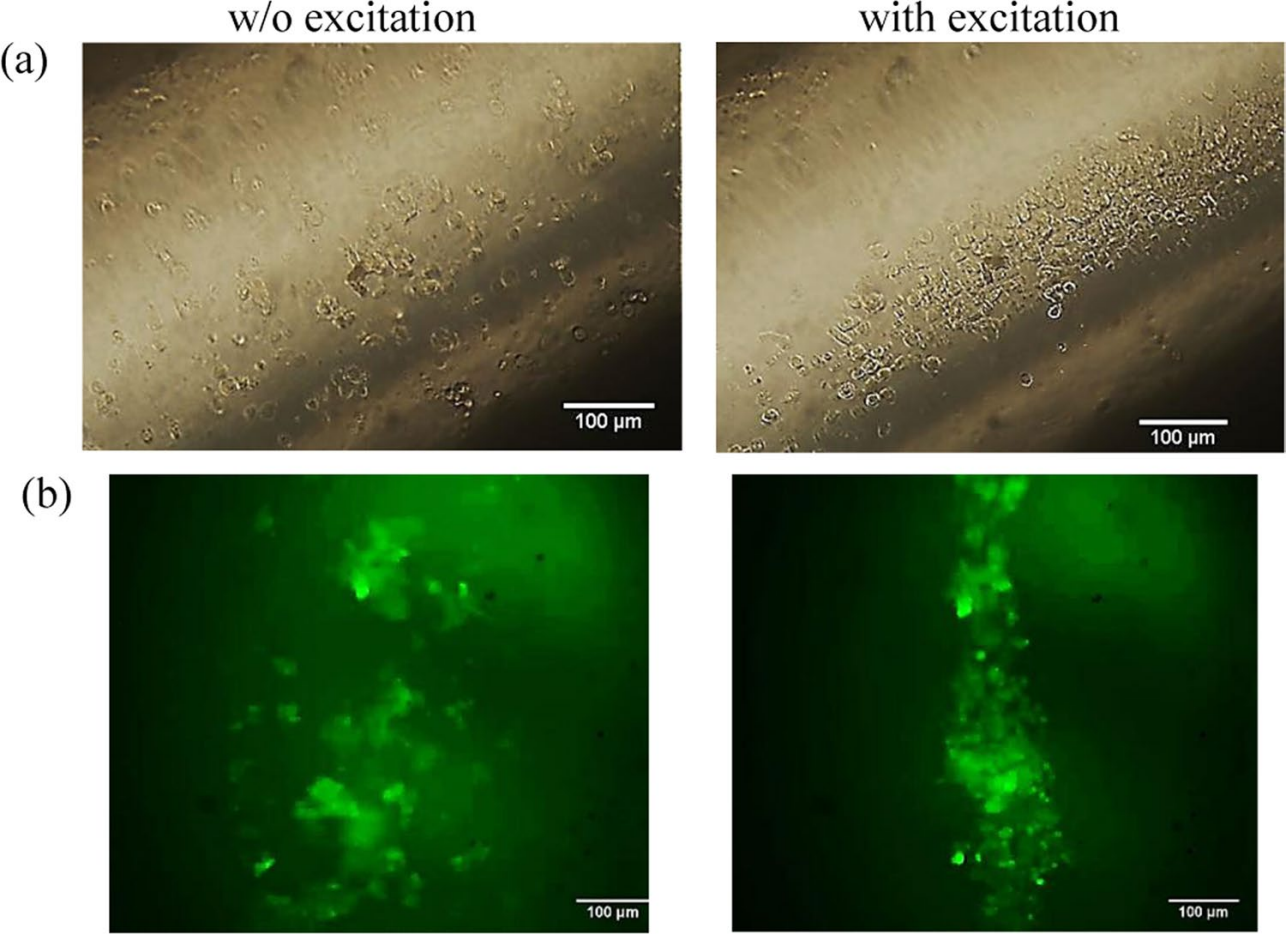
Representative (**a**) bright-field and (**b**) fluorescent figures of HUVECs distribution in the cylindrical nozzle without (left) and with (right) acoustic excitation at 877 kHz from the side view, scale bar presenting 100 μm.

The resonant frequency of structural vibration of the cylindrical glass tube was numerically and experimentally determined as 871 kHz and 877 kHz, respectively. Cells suspended in the fluid were accumulated towards the center of the glass tube with acoustic excitation both in the bright field and under fluorescent microscope (see Fig. 1). It is noted that the timing of microparticle and cell to reach a pressure node is different. The accumulation of 6-µm microparticles towards the center of the cylindrical tube took about 0.73 s. However, it took 1.66 s for 6-µm C2C12 cells to reach the center of the cylindrical tube. Such a discrepancy is due to that the compressibility of the microparticle is half of that of the cell^26^. Hence, acoustic radiation force acting on the cells is lower in magnitude.

**Figure 2:**
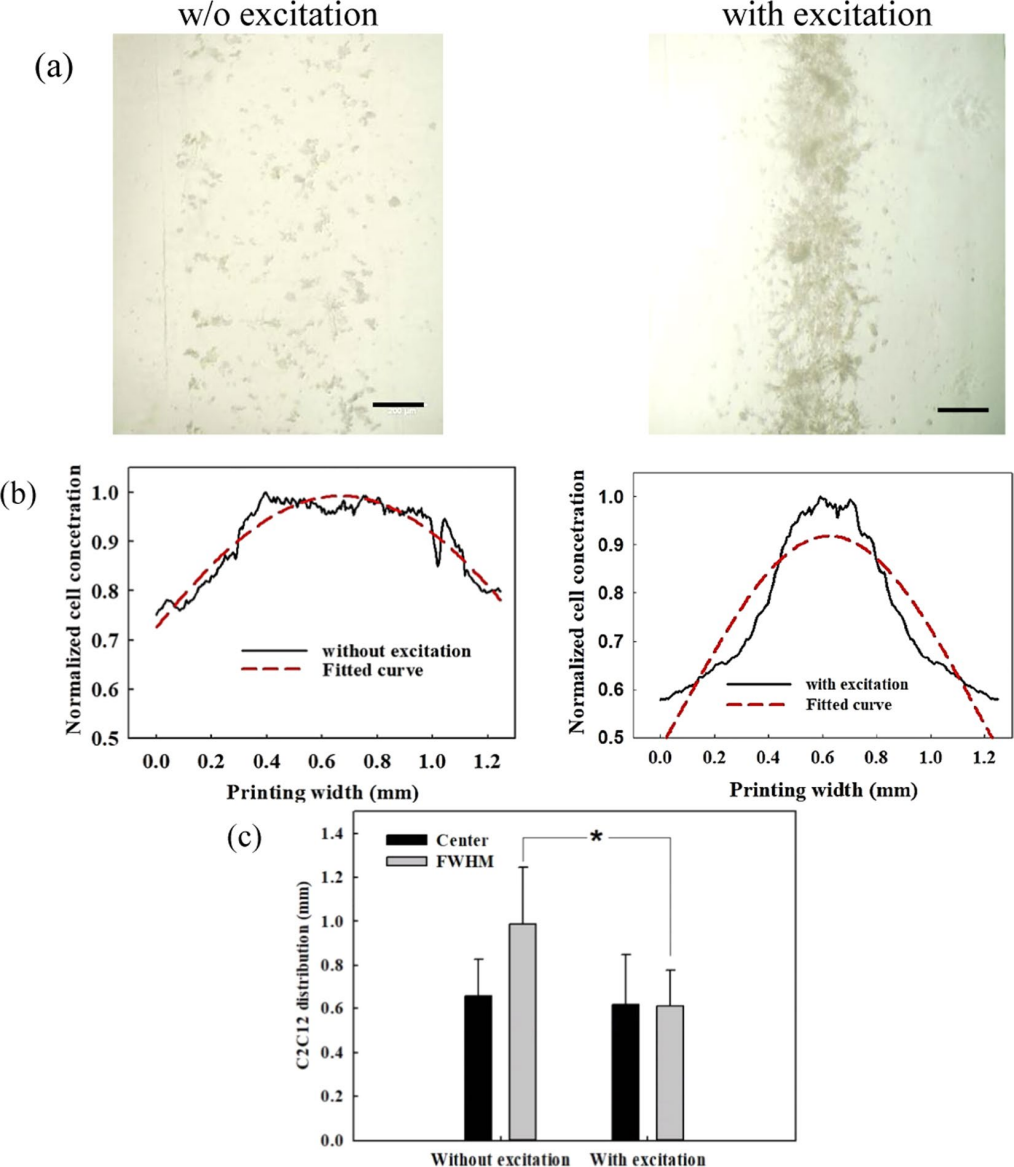
Representative micrograph of (**a**) C2C12 cells in the printed construct of 5% GelMA without and with acoustic excitation, scale bar presenting 200 μm, (**b**) the corresponding cell distribution fitted by a Gaussian curve in dashed line, and (**c**) comparison of the center and FWHM of the spatial cell distribution from fitted Gaussian curves (*n* = 6), *significant difference (*p* < 0.05)^61^.

The acoustic excitation was found to have little changes to the shape of the printed construct on the 4″ petri dish. The construct had a width of about 1.0–1.3 mm, and the distribution of cells in it was quantitatively analyzed (see Fig. 2). It was found that the cell spreading was fairly random and uniform without acoustic excitation. The center and FWHM of C2C12 cell distribution fitted by the Gaussian curve are 0.66 ± 0.17 mm and 0.98 ± 0.26 mm, respectively. In contrast, the cells undergone acoustic excitation spread densely at the center but sparsely near the edge of the printed construct. The corresponding center and FWHM are 0.62 ± 0.23 mm (*p* = 0.739) and 0.61 ± 0.17 mm (*p* = 0.015), respectively. Furthermore, the printing of HUVECs has similar performance. The center and FWHM of HUVECs cell distribution without and with acoustic excitation are 0.84 ± 0.26 mm vs. 0.69 ± 0.19 mm (*p* = 0.281) and 1.64 ± 0.37 mm vs. 1.07 ± 0.21 mm (*p* = 0.008), respectively. Overall, acoustic excitation could accumulate the cells at the center of printed constructs no matter of the cell types.

### Cell viability and morphology

**Figure 3:**
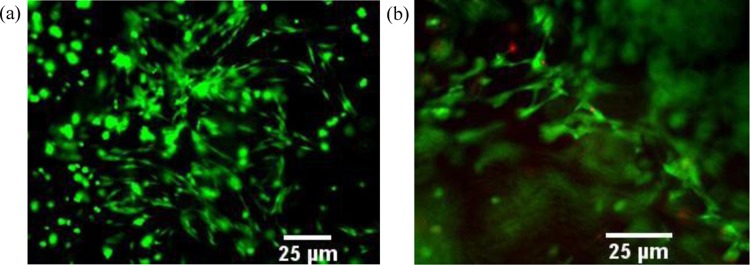
Representative photos of live-dead staining (**a**) C2C12 cells and (**b**) HUVECs in 5% GelMA with acoustic excitation, scale bar of 25 µm^61^.

**Figure 4:**
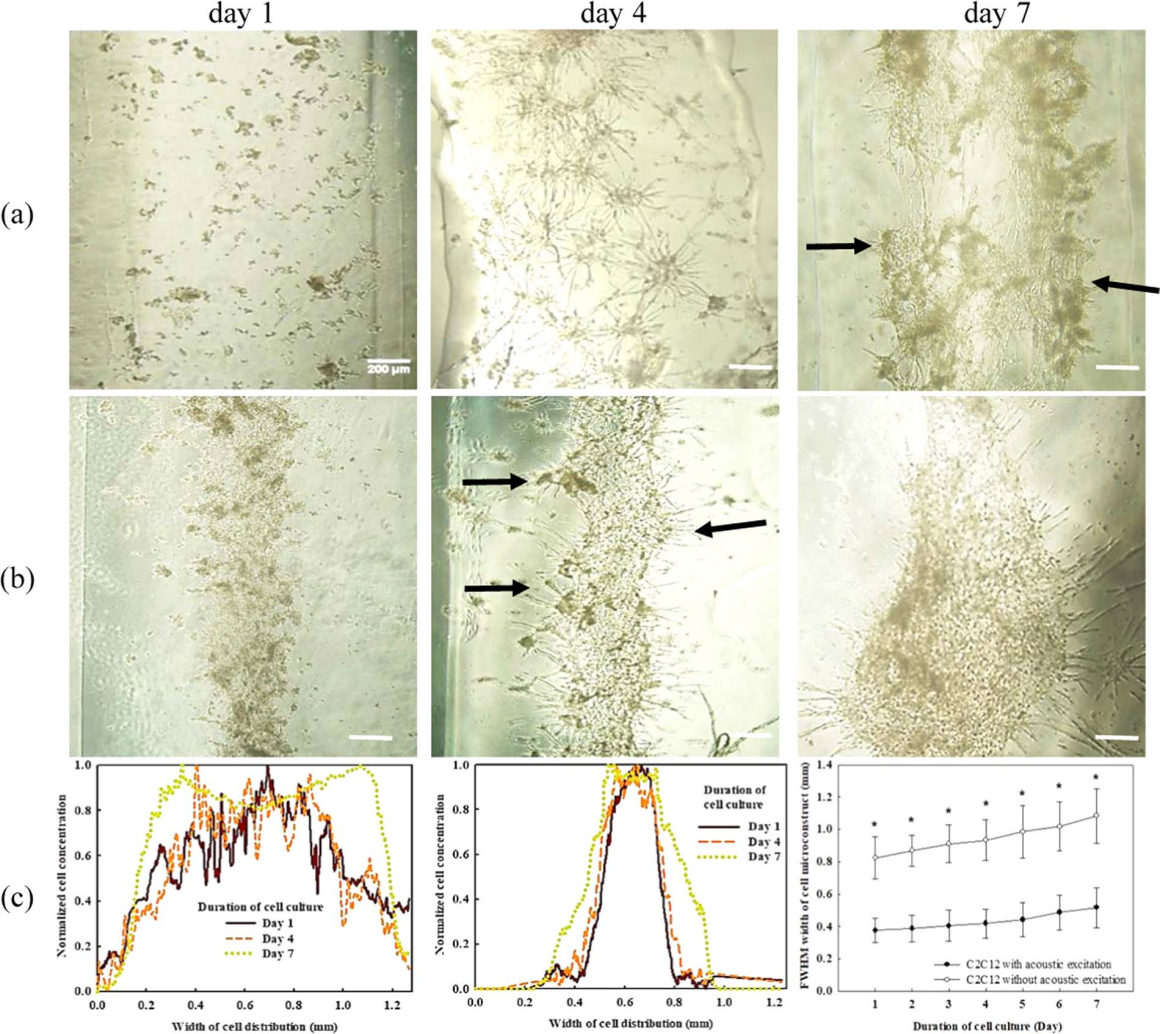
Representative photos of C2C12 cells in microscopy (**a**) without and (**b**) with acoustic excitation on day 1, day 4, and day 7, scale bar presenting 200 µm, and **(c**) comparison of the spatial cell distributions (left: without acoustic excitation, middle with acoustic excitation) and cell growth during culturing in GelMA, arrows showing the formation of myotube, *statistical difference (*p* < 0.05)^61^.

The performance of cells after the bioprinting was evaluated. Cell viability of C2C12 and HUVECs are 95.1 ± 3.6% and 89.4 ± 4.8%, respectively, by using the live-dead staining kit as shown in Fig. 3. The corresponding values for the control group (printing without acoustic excitation) are 95.9 ± 3.8% and 91.0 ± 5.4%, respectively. No significant differences (*p* = 0.741 and 0.634 for C2C12 and HUVEC, respectively) between these two groups suggest that acoustic excitation has little influence on the cell viabilities. The distributions of cells without and with acoustic excitation were monitored for up to 7 days (see Fig. 4). On day 4, the cells without acoustic excitation initially spread thoroughly the whole printed construct and then grew individually without a significant connection between them. On day 7, the cells grew further and showed compaction of the structure due to the cell differentiation (see arrows in Fig. 4a). The FWHM of the printed cells increased from 0.82 mm on day 1 to 1.08 mm on day 7. In comparison, the cells undergone the acoustic excitation showed a distinct dense cell distribution at the center of the printed construct. On day 4, the connection with the adjacent cells had already been established. Some cells could even sprout outward from the center of construct where the cells accumulate densely (see arrows in Fig. 4b). The width of printed cells undergone the acoustic excitation increased from 0.38 mm on day 1 to 0.52 mm on day 7 but still significantly lower than that without acoustic excitation (*p* < 0.001). HUVECs in the printed construct showed the similar performance, higher accumulation at the center of the printed construct with acoustic excitation and, subsequently, more sprouting from HUVECs cocultured with fibrin as a supporting structure for attachment and elongation (data not included, but neovasculature data will be shown later).

### Orientation of C2C12 cells

**Figure 5:**
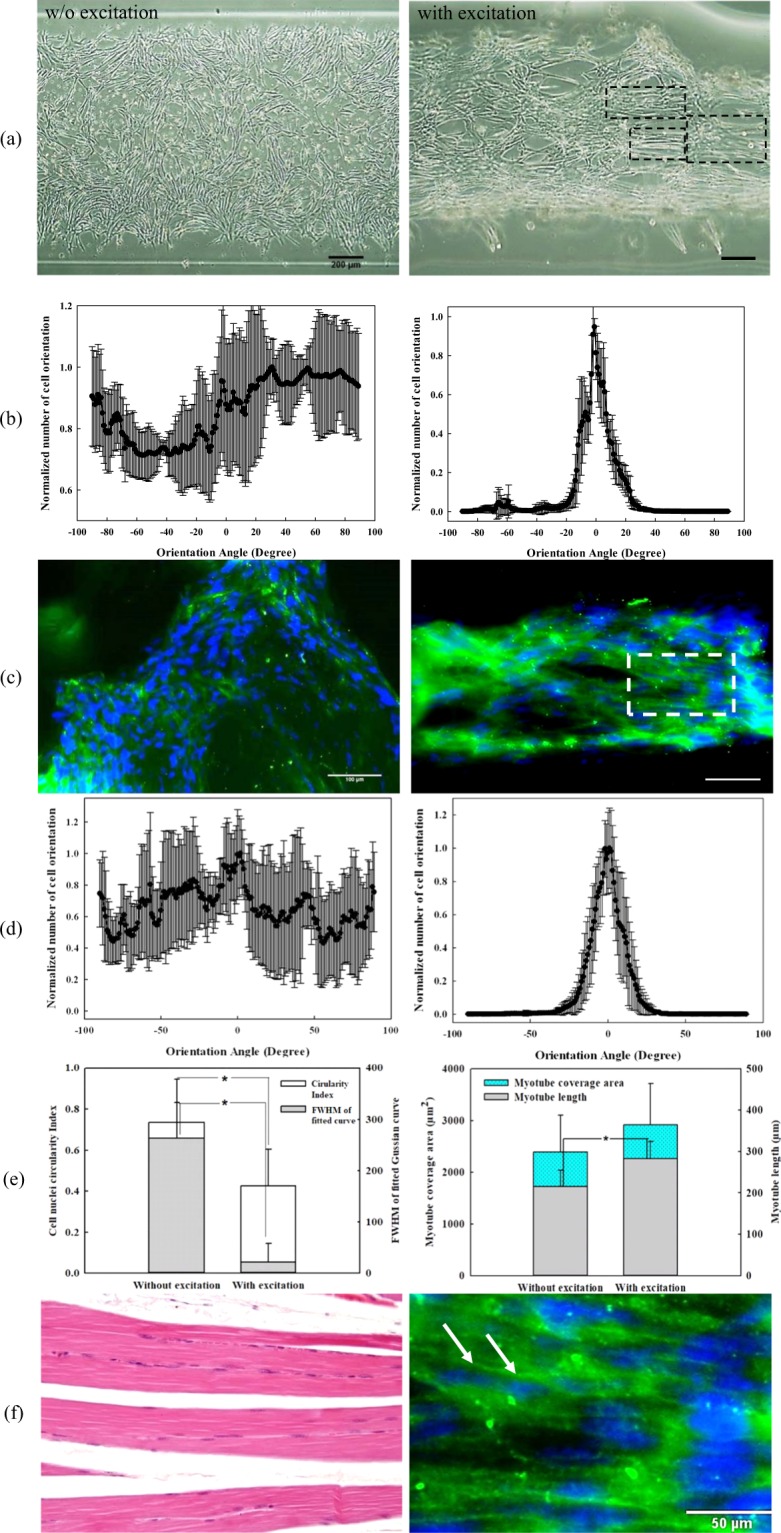
(**a**) Phase-contrast micrograph of alignment of C2C12 in the GelMA construct on day 7, scale bar of 200 µm, dashed rectangles show cell fusion and muscle cell bundles, (**b**) normalized histogram of cell orientation in the contrast microscopy represented in the format of mean ± std, (**c**) immunofluorescence (IF) against myosin heavy chain (green color) and cell nuclei counterstained by DAPI (blue color) of the printed construct, scale bar of 100 µm, (**d**) normalized histogram of cell nuclei orientation in the immunofluorescent imaging, (**e**) FWHM of fitted Gaussian curve and cell nuclei circularity, *statistical difference (*p* < 0.05), and (**f**) histological comparison of skeleton muscle alignment in human tissue^34^ in the left and zoomed-in area obtained from the white window of (**c**) in the right, arrows show the orientation of the C2C12 muscle fibers, scale bar of 50 µm^61^.

The orientation of C2C12 cells was determined using both phase-contrast micrograph and immunofluorescent staining for quantitative comparison (see Fig. 5). C2C12 cells without acoustic excitation grew and spread out thoroughly into the GelMA construct within 5 days of cell culture in random directions. In contrast, the accumulated C2C12 cells at the center of the printed construct with acoustic excitation show a strong tendency of alignment along the printing direction, the majority of them (> 90%) being aligned from −30° to 30° with the peak at 0° (see Fig. 5b on the right). In addition, the phase-contrast microscopy has also revealed that the partial formation of muscular cell syncytia in the constructs indicated by the cell fusion and formation of short muscle cell bundles (see dashed rectangles in Fig. 5a).

C2C12 cells were laden in GelMA construct during the process of acoustic excitation, printing, UV curing, and immunofluorescence (IF) staining. Fluorescent micrographs of the differentiated C2C12 cells show cell nuclei in blue and myosin heavy chain in green that were counterstained with DAPI and the MHC-immunofluorescence, respectively. With an MHC-IF signal, the orientation of C2C12 cell nuclei was analyzed. The cell-to-cell syncytium was not clearly observed throughout the printed construct without acoustic excitation. The orientation of cell nuclei distributes quite uniformly from −90° to 90°. In contrast, most of the cells undergone the acoustic excitation align parallel to form the tandems of muscular fascicles. Their combined MHC and DAPI stain, especially the magnified area, unveils similar orientation of the C2C12 muscle fibers as the natural skeletal muscle fibers^34^ (see Fig. 5f). The normalized histogram of cell nuclei orientation shows an apparent Gaussian curve from −30° to 30° with the peak at 0° (see Fig. 5d), which is similar to the observation under the phase-contrast microscope.

FWHM of the cell distribution and the circularity of cell nuclei in the printed construct with acoustic excitation are significantly lower than that without excitation (*p* < 0.01 in Fig. 5e). The length of myotube with acoustic excitation on day 7 is significantly longer than that without acoustic excitation (282.2 ± 42.5 µm vs. 215.0 ± 39.2 µm, *p*  =  0.017). But myotube coverage area under both conditions are similar (2921.1 ± 792.3 µm^2^ vs. 2389.9 ± 709.4 µm^2^, *p* = 0.249).

### Neovascularization of HUVECs

**Figure 6:**
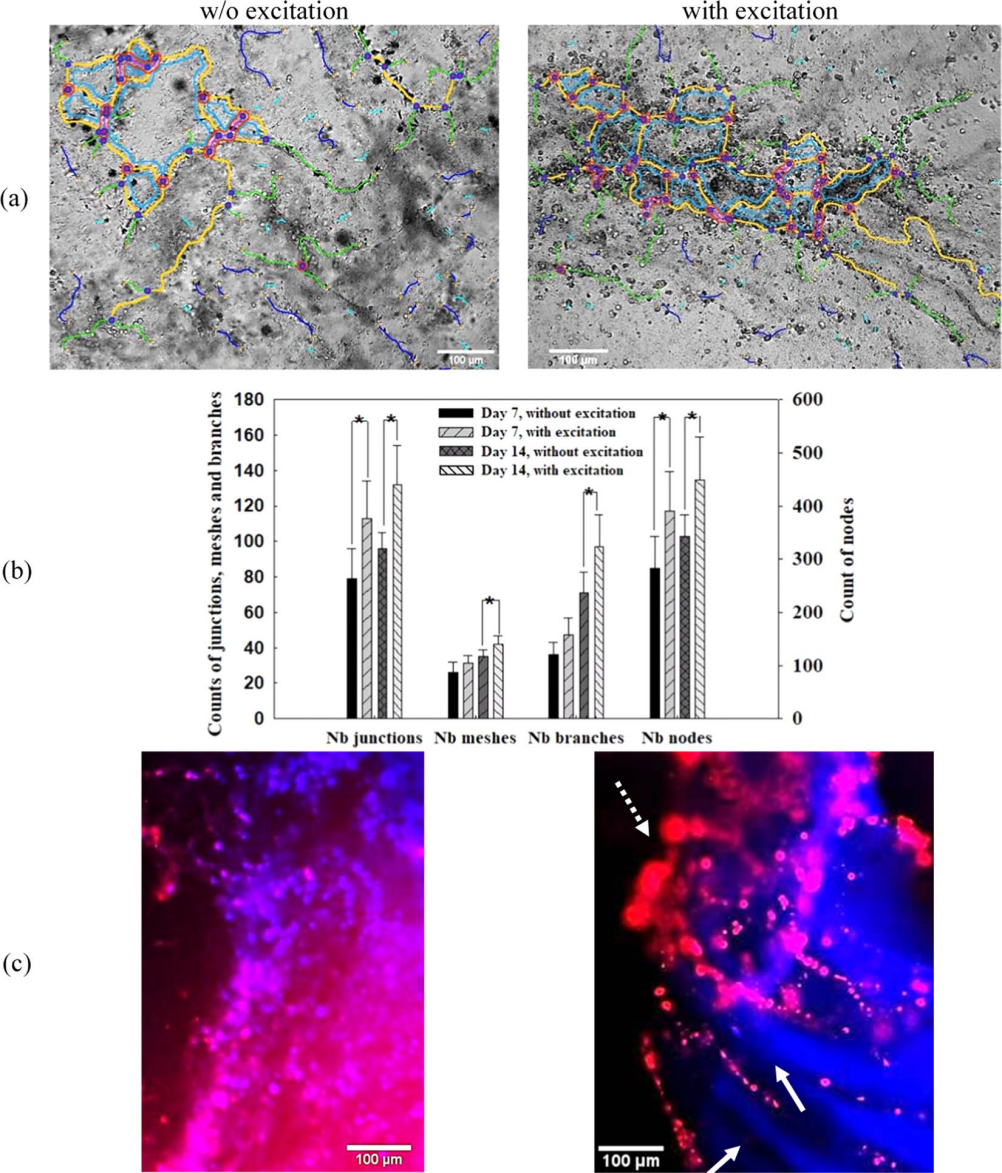
(**a**) Representative microscopic image of HUVECs with detected nodes, junction, meshes, and branches on day 14, scale bar presenting 100 µm (**b**) comparison of the number of nodes, junctions, meshes, and branches of HUVECs in GelMA-fibrin on day 7 and 14, *statistical difference (*p* < 0.05) and (**c**) fluorescent image of HUVECs stained by CD31 and DAPI, dashed arrow shows the main branch of aggregated HUVECs and solid arrows show small branches of HUVECs stretching out from the main branch on day 14, scale bar presenting 100 µm.

The characteristics of the neovascularization from HUVECs in the printed GelMA-fibrin construct, such as the number of nodes, junctions, meshes, and branches, were analyzed on day 7 and 14 (see Fig. 6). The numbers of nodes and junctions of the printed HUVECs with acoustic excitation are statistically higher (*p* = 0.031 and 0.011, respectively) than those without acoustic excitation. However, the numbers of meshes and branches of the printed HUVECs GelMA construct between these two groups show statistical difference (*p* = 0.038 and 0.027, respectively) only on day 14 but not on day 7, which suggests that our HUVECs in GelMA-fibrin may need about 1–2 weeks to form the linkage with the adjacent cells and establish branches. Additionally, the CD31 in red and DAPI in blue of the HUVECs cells in the printed construct without and with acoustic excitation are shown in Fig. 6c. HUVECs with acoustic excitation enhanced the formation of both main branch (dashed arrow) and sub-branches (solid arrow). In contrast, few continuous and long branches were found from the scattered HUVECs without acoustic excitation in the printed construct on day 14.

## Discussion

The patterning of cells using the acoustic excitation was investigated in this work. There is a good agreement between the numerical simulation and experimental measurement of the resonant frequency (871 kHz vs. 877 kHz). The slight discrepancy between them may be due to the inconsistent tube diameter and material properties. The distribution of printed cells undergone the acoustic excitation has significant narrow FWHM width, similar to the pattern observed in the cylindrical tube by the structural vibration. However, the center of cell distribution between both groups has only a slight difference (~0.06 mm). Subsequently, the cells were monitored during the culture process for their growth, morphological changes, and differentiation. The width of accumulated C1C12 cells throughout the printed construct initially expanded over time but gradually became saturated which is usually found in the cell differentiation in the confined space^35–37^. Thus, the cell accumulating characteristics were compared on day 7. The orientation of the cells under contrast microscope and cell nuclei under fluorescent microscope showed that most of them undergone the acoustic excitation elongated along the printing direction both before and after cell differentiation. Since C2C12 cells are the skeletal myoblast cell line, their dense package expedites the differentiation to the muscle fascicles because of the enhanced cell-to-cell interaction for skeletal muscle morphogenesis^38,39^. In addition, the lower value of cell nuclei circularity suggests that the cell orientation is along the major axis with acoustic excitation to promote the elongation and orientation^40^. The numbers of nodes, junctions, meshes, and branches are used to quantitatively evaluate the neovascularization of HUVECs^41,42^. Node and junction usually occurred at the original location of HUVECs after printing, illustrating the connection from at least three segments. The accumulation of HUVECs potentially enhances the growth and formation of capillary-like structure. Encapsulated HUVECs in the scaffold require the molecules released from nearby cells to proliferate and function^43–45^. Meanwhile, branches and meshes represent a structural connection between each HUVEC nodes and junctions. Their numbers with acoustic excitation only become significantly larger on day 14, which may be because the process of capillary-like formation takes a few weeks in the hydrogel^46^.

High-resolution cell engineering is essential to replicate complex and highly ordered tissues *in vivo*, but achieving such a goal by bioprinting, especially using the extrusion-based printer, is challenging because of the limited nozzle and droplet sizes. Acoustic excitation of the nozzle could be applied to various types of cells. The primary cells derived from the human organs (e.g. liver, heart, and skin) could be further tested. Embryonic stem cells (ESCs), adult stem cells (ASCs), induced pluripotent stem (iPS) cells, and tissue-specific cell line to the desired phenotype would enhance the immune acceptance for *in vivo* safety and efficacy. To further improve the cell manipulation (e.g. faster motion and denser accumulation) greater acoustic radiation force will be utilized by increasing the acoustic power. Another potential of this approach is to selectively accumulate different types of cells at various positions for co-culture, which is important in producing artificial tissues under *in vitro* conditions. The magnitude of acoustic radiation force acting on the cells is proportional to their volumes. Hence, large cells will be densely packed into single or multiple lines at the pressure node while leaving small cells scattered randomly in the printed construct. For instance, a human blood vessel in the dermis is grown from endothelial cells (~10 μm for HUVECs) surrounded by groups of fibroblasts (~4 μm), pericyte, and muscle cells. Due to the size difference (~2.5 fold), the acoustic radiation force applied to fibroblasts is ~15 fold lower than HUVECs. Co-aligned HUVECs and human adipose-derived stem cells (hADSCs) that are arranged in a biodegradable catechol-conjugated hyaluronic acid (HA-CA) hydrogel exhibit the enhanced cell-cell contacts, upregulated gene expression of Tie2 and von Willebrand factor (vWF), the expression of a mural cell marker [smooth muscle alpha-actin (α-SMA)] in hADSCs, and secretion of angiogenic and anti-inflammatory paracrine factors (e.g. VEGF and IL-10) for enhanced angiogenesis and decreased apoptosis at ischemic defect sites^31^. Co-culture of endothelial and stromal cells promoted the formation of homogeneous microvessels by inducing the self-organized capillaries^14,16^.

The striated myofibers (myocytes) consist of the arrays of thick myosins parallely alternated and interdigitated with actin myofilaments along the length, which makes the striation of muscle fibers. The differentiation of C2C12 cells is compulsorily undergoing in the direction of striated myocyte development upon a specific activation. Myoblasts are destined to take the elongated geometry so as to survive and maintain parallel actin filaments along the stretching direction, which are the prerequisites for the normal functions of muscle cells. Mechanical stretch is a key factor that determines the optimal geometry of myoblast C2C12 cells under stretch whereas vascular endothelial cells and fibroblasts had no such dependency^47^. In narrower confinement (e.g. microchannel), C2C12 cells show a better orientation^36^. Similarly, cellular alignment is highly dependent on the line width of the printed construct. At the linewidth of 500 µm and high cell density of 5 × 10^6^ cells/mL, most of the cells (64 ± 9%) were oriented within 10° in the construct, while those with a line width of 5000 µm showed randomized cell orientation^31^. However, a thorough understanding of this phenomenon of geometrical confinement is still limited. Small nozzle tip and high cell density may also cause the nozzle clogging, which seriously affects the accuracy and reliability of nozzle-based printing and damages the nozzle. Furthermore, a shear force can be generated at the nozzle that may induce damage to the cell and decrease cell viability during printing. Cell viability was affected by the flow rate, material concentration, dispensing pressure, and nozzle geometry. Sufficiently high viscosity is essential for the biomaterial suspension to overcome the surface tension-driven droplet formation and be drawn in the form of straight filaments. On the other hand, it triggers the nozzle clogging and should be optimized. Using a large nozzle with acoustic excitation may solve such problem, confining the cells in a small linewidth and minimizing the nozzle clogging simultaneously. Cell viability is a critical issue in the bioprinting. The high values of C2C12 and HUVECs with acoustic excitation (95.1 ± 3.6% and 89.4 ± 4.8%, respectively, on day 14) suggest that acoustic manipulation approach at low power output has little influence. In a previous study, *Petunia hybrid* cell suspensions under the 20-min sonication of the standing wave at 2.15 MHz and energy density of 8.5 J/m^3^ (pressure amplitude of 0.28 MPz) has similar cell viability as the control group (~95%)^48^.

Low-intensity ultrasound can significantly improve angiogenesis either *in vitro* or *in vivo* at an intensity of *I*_*SATA*_ = 0.05–0.4 W/cm^2^ at the frequency of 45 kHz-3 MHz^49–51^. The angiogenesis-related cytokines, IL-8 and fibroblast growth factor (bFGF), were significantly stimulated in osteoblasts, and VEGF was significantly stimulated in human mandibular osteoblasts, gingival fibroblasts and peripheral blood mononuclear cells (monocytes)^52^. Meanwhile, ultrasound exposure can enhance the proliferation and differentiation of many types of cells, such as mesenchymal stem cells (MSCs)^53^, odontoblast-like cells^54^, C2C12 cells^55^, and hematopoietic stem/progenitor cell^56^. Cell motility is also promoted by ultrasound due to the induced Rac activation associated with dramatic actin cytoskeleton rearrangements^57^. Therefore, acoustic excitation in the nozzle may benefit the growth, maturation, and diffusion in the printed tissue samples without compromising the cell viability if the power and induced temperature elevation by the piezoceramic plate are in good control. The influence of ultrasound exposure on the angiogenesis will be investigated later.

In the future work, the printed constructs will be cultured for a longer time (~30 days) for maturation. Functionalities of the neovascular network (e.g. diffusion of blood and oxygen, and disposal of waste) will be assessed. Preparation of three dimensional sample (e.g. stacking of multiple two-dimensional constructs) with various cell types is an important step in tissue engineering.

## Conclusions

Effective and quick patterning of biological cells in the printed construct using the structural vibration of a cylindrical glass tube was proposed and evaluated in this work. The distribution of C2C12 cells with acoustic excitation has a significantly narrow FWHM cell width in the 5% GelMA construct than that without acoustic excitation (0.38 ± 0.08 mm vs. 0.82 ± 0.13 mm, *p* < 0.001). The acoustically-excited cells establish strong cellular connections and elongate in the printing direction. Immunofluorescent staining indicates a greater alignment/orientation of cell nuclei and myosin heavy chain in the differentiated C2C12 cells. The zoomed-in figure unveils the morphological and histological similarity of these acoustically-excited C2C12 muscle fibers to the natural skeletal muscle fibers. The performance of acoustic excitation on HUVECs is similar, narrow FWHM cell width (0.41 ± 0.1 mm vs. 0.92 ± 0.21 mm, *p* < 0.001). Significant angiogenesis was found in the accumulated HUVECs around fibroblasts. The number of nodes and junctions per area formed with acoustic excitation showed a significant increase (282.3 ± 61.8 vs 407.5 ± 75.4, *p* = 0.010, and 79.5 ± 17.5 vs 113.5 ± 19.7, *p* =  0.011, respectively). Furthermore, the main branch of HUVECs with acoustic excitation could be formed successfully at the center of the printed construct on day 14. Overall, acoustic excitation is a convenient, cost-effective, and biocompatible method for patterning and accumulation of cells. The accumulated cells along the printing can enhance cell elongation and differentiation for the tissue maturation and functionality.

## Materials and Methods

### Preparation of bioink

Two types of cells, C2C12 cells and HUVECs, were used for the bioprinting in this study. C2C12 cells, an immortalized mouse skeletal myoblast cell line (CRL-1772™, ATCC®), were cultured in HyClone^TM^ Dulbecco’s modified eagle’s medium (DMEM, GE Healthcare Life Sciences, HyClone Laboratories), which contained 10% fetal bovine serum (FBS, Gibco) and 1% antibiotic-antimycotic solution, including 10,000 units/mL of penicillin, 10,000 µg/mL of streptomycin, and 25 µg/mL of amphotericin B (Gibco), in a cell culture flask (t75, Thermo Fisher Scientific). At the confluence of 80–90%, C2C12 cells were harvested with the standard trypsinization. Briefly, the cells were incubated with 0.25% trypsin-1mM EDTA solution (Lonza) at 37 °C for 3 minutes. The reaction of trypsin was terminated with a cell culture medium for 5 minutes at room temperature (25 °C). The cells were then washed with 1× phosphate-buffered saline (PBS, Sigma-Aldrich) and centrifuged at 1,000 RPM (SL 8, Thermo Fisher Scientific) for 5 minutes.

HUVECs (C-12200, PromoCell) after passage 6 were cultured in endothelial cell basal medium-MV (C-22220, PromoCell) mixed with 1% antibiotic-antimycotic solution, including 10,000 units/mL of penicillin, 10,000 µg/mL of streptomycin, and 25 µg/mL of amphotericin B (Gibco), in the cell culture flask. The cells were incubated in a humidified incubator (Heracell 150i, Thermo Fisher Scientific) at 37 °C and 5% CO_2_. The culture medium was changed every two days. Achieving 90% confluence, the cells were dissociated using 0.25% Trypsin 1 mM EDTA.4Na (Lonza) and centrifuged at 1,000 RPM for 5 min at room temperature. The initial cell concentration was enumerated using a standard hemocytometry (79001-00, Cole-Parmer).

GelMA at the concentration of 5% was prepared using the established protocol^58^. Then, a freeze-dried foamy GelMA was dissolved in DMEM. The GelMA solution was mixed with 0.2 g of a photoinitiator (Irgacure 2959, Sigma-Aldrich) and then kept in a dark chamber at 37 °C till use. The cells (C2C12 cells or HUVECs) were finally embedded in 2 mL of 5% GelMA at the concentration of 2.0×10^6^ cells/mL for bioprinting.

### Experimental setup of bioprinting

**Figure 7:**
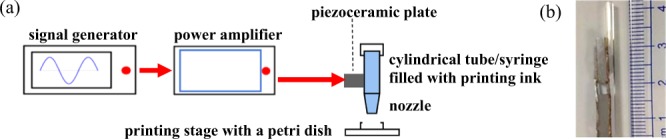
(**a**) Schematic diagram of the experiment setup and (**b**) photo of two piezoceramic plates attached to a glass nozzle for bioprinting.

Two piezoceramic plates (APC International) in a dimension of 2.0 mm × 1.0 mm × 0.5 mm was connected in parallel and glued (3M Scotch-Weld EC40) to the glass tube with the inner and outer diameter of 0.8 mm and 1.0 mm, respectively. The sinusoidal signal generated from a function generator (AFG3000, Tektronix) at the resonance frequency of 877 kHz was amplified by a power amplifier (240 L, ENI) before delivering to a lab-made impedance matching unit and the piezoceramic plates. The schematic diagram of the experimental setup is illustrated in Fig. 7. The mixture of C2C12 cells or HUVECs-embedded GelMA was loaded in a 10 mL syringe and then printed on a 4-inch petri dish using an extrusion-based printer (TechnoDigm) at a stage speed of 100 mm/min. To activate C2C12 cell differentiation after the bioprinting, the printed construct was incubated with 10% horse serum (H0146, Sigma-Aldrich) in DMEM while the printed construct with HUVECs was incubated with HUVEC culture medium mixed with fibroblast-conditioned medium (FCM) in a humidified incubator with 5% CO_2_ at 37 °C. The culture medium was changed every three days. The fibroblast-conditioned medium was retrieved from fibroblast growth medium-2 (FGM-2, Lonza) after 2 days of fibroblast culture (NHDF-Ad, Lonza) at a concentration of 5 × 10^5^ cells/mL. The culture process was up to 14 days.

### Evaluation of cell distribution and alignment

For both experiments of without and with acoustic excitation, the distribution of both cells in the printed construct was observed under a light microscope (CKX-41, Olympus) immediately after the extrusion-based printing and during the cell culture process, and then the captured images were quantitatively analyzed using digital image processing software (ImageJ, National Institute of Health) and computational software (MATLAB, MathWorks) to determine its features, such as the spatial cell concentration, width of cell-laden construct, coverage area, cell circularity, and cell nuclei orientation. The histogram of deposited cells in the printed construct was fitted by a Gaussian function

1 $$f(x)=\frac{1}{\sqrt{2\pi {\sigma }^{2}}}{e}^{-\frac{{(x-\mu )}^{2}}{2{\sigma }^{2}}}$$

where *μ* is the mean value, and *σ* is the standard deviation. The corresponding full width at half maximum (FWHW = 2.355 · *σ*) in the Gaussian curve was used to evaluate the width of cell distribution and compare the performance of acoustic excitation in the bioprinting.

The cell orientation and circularity were quantified using OrientationJ-plugin in ImageJ (National Institute of Health) which automates the digital image analysis in a region-of-interest (ROI) based on the evaluation of the structure tensor in a local neighborhood. The image contrast was adjusted appropriately in order to clearly distinguish the cell boundary and nuclei. Cell nuclei alignment was determined from DAPI (4’,6-diamidino-2-phenylindole, D9542, Sigma-Aldrich) signal. The cell circularity was calculated based on the following equation

2 $$4\pi A/{p}^{2}$$

where *A* is an area of the cell, and *p* is a perimeter of the cell. The representative photos of the cell-laden construct were taken every 3 days.

### Viability and morphological analysis of C2C12

Cell viability of C2C12 was quantified through a commercially available live-dead staining kit (04511-1KT-F, Sigma-Aldrich). Briefly, the cell-laden constructs were exposed to a solution consisting of calcein (0.5 µL/mL) and ethidium homodimer (ETD, 2 µl/ml). Following the viability staining, the constructs were rinsed three times and imaged by the fluorescent microscope (Axio Vert.A1, Carl Zeiss). The captured images were automatically analyzed using a built-in script in the ImageJ, which uses particle segmentation to pick out the live and dead cells, respectively.

For the morphometric analysis, myotubes were considered multinucleated muscle fibers containing at least three nuclei^59^. The length, coverage area, circularity, and nuclei orientation of the myotubes were analyzed using ImageJ and averaged from at least 6 random areas per sample^60^. The nuclei orientation of each cell in the GelMA construct was assessed and represented in the format of the histogram. A circularity value of 1.0 indicates a perfect circle. As this value approaches 0.0, it indicates an increasingly elongated polygon.

### MHC-immunofluorescence of C2C12 and HUVECs

To increase the permeation of printed GelMA to the antibody, the constructs were incubated with cool IFPerm III^®^ solution, which consists of 2 mL of acetone, 1 mL of methanol, 0.5 mL of formalin, 0.5 mL of Tween 20, and 0.25 mL of Triton X (all purchased from Sigma-Aldrich), at 4 °C for 2 days. They were washed twice with PBS and desiccated. They were then incubated with anti-human myosin heavy chain (MHC) antibody [clone MF 20, 1:10, developmental studies hybridoma bank (DSHB)] at 4 °C for 2 days and washed twice with 1× PBS prior. The constructs were then incubated with an anti-human immunoglobulin-G antibody (Sigma-Aldrich) labeled with FIT-C (fluorescein-5-isothiocyanate, 1:50, Sigma-Aldrich) at 4 °C for 1 day. They were washed twice with PBS and counterstained with DAPI. Immunofluorescence was acquired under the fluorescent microscope (Axio Vert.A1, Carl Zeiss) with red, blue and green filters. For HUVECs, 1/100 dilution of mouse monoclonal anti-CD31 antibody (Thermo Fisher Scientific) in a total volume of 200 µL was stained overnight.

### Angiogenic analysis of HUVECs

In suitable culture conditions, HUVECs could form structures that can branch and mimic a capillary-like *in vitro*. A number of nodes, junctions, meshes, and branches developed from 5% HUVEC-contained GelMA construct with fibrin^41,42^. This formation of the capillary-like structure was quantitatively evaluated using the toolbox of angiogenesis analyzer in ImageJ.

### Statistical analysis

At least 6 samples in these experiments (C2C12 and HUVECs) were used for the statistical analysis using SigmaPlot (Systat Software). The student *t*-test was used for statistical analysis with 95% confidence interval.
